# Star-Like Thermoresponsive Microgels as an Emerging Class of Soft Nanocolloids

**DOI:** 10.1021/acsnano.5c08174

**Published:** 2025-09-29

**Authors:** Elisa Ballin, Francesco Brasili, Tommaso Papetti, Jacopo Vialetto, Michael Sztucki, Simona Sennato, Marco Laurati, Emanuela Zaccarelli

**Affiliations:** ‡ Dipartimento di Fisica, Sapienza Università di Roma, Piazzale A. Moro 5, 00185 Roma, Italy; § CNR-ISC, Uos Sapienza, Piazzale A. Moro 5, 00185 Roma, Italy; ⊥ Dipartimento di Chimica “Ugo Schiff”, Università di Firenze, 50019 Sesto Fiorentino (FI), Firenze, Italy; ∥ Consorzio per lo Sviluppo dei Sistemi a Grande Interfase, via della Lastruccia 3, 50019 Sesto Fiorentino (FI), Firenze, Italy; ¶ 55553 European Synchrotron Radiation Facility (ESRF), 71 avenue des Martyrs CS40220, 38043 Grenoble, Cedex 9, France

**Keywords:** star polymers, microgels, soft colloids, form factors, monomer-resolved simulations

## Abstract

We provide experimental and numerical evidence of an emerging class of soft nanocolloids: star-like microgels with thermoresponsive character. This is achieved by using the standard precipitation polymerization synthesis of poly­(*N*-isopropylacrylamide) (PNIPAM) microgels and replacing the usually employed cross-linking agent, *N*,*N*′-methylenebisacrylamide (BIS), with ethylene glycol dimethacrylate (EGDMA). The fast reactivity of EGDMA, combined with its strong tendency to self-bind, produces colloidal networks with a central, cross-linker-rich core, surrounded by a corona of long, cross-linker-free arms. These star-like microgels fully retain PNIPAM thermoresponsivity and undergo a volume phase transition at a temperature of ∼32 °C that is very sharp compared to standard PNIPAM–BIS microgels, independently of the cross-linker content. Dynamic light scattering and small-angle X-ray scattering experiments are compared to extensive simulation results, based on ideal star polymers as well as on state-of-the-art monomer-resolved simulations, offering microscopic evidence of the star-like internal structure of PNIPAM–EGDMA microgels. This can be described by an appropriate model for the form factors combining star and microgel features. The present work thus bridges the fields of star polymers and microgels, providing the former with the ability to respond to temperature via a facile synthetic route that can be routinely employed, opening the way to exploit these soft particles for a variety of fundamental studies and applicative purposes.

## Graphical abstract

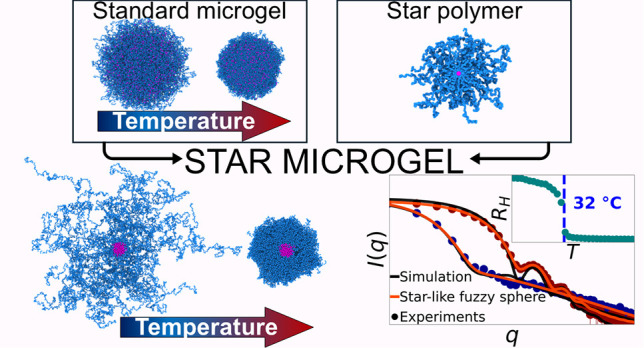

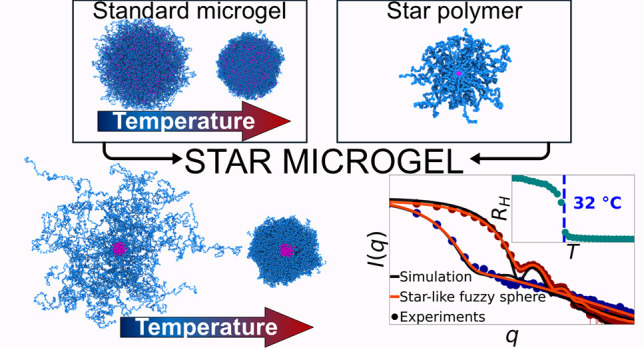

## Introduction

Star polymers are nanocolloids made of multiple arms radiating from a central core; this class of polymers has gathered in the past significant attention both for their fundamental theoretical interest^1,2^ and for their potential applications in various fields, including biomedicine, environmental remediation, and advanced materials, because of the large spectrum of functionalizations available for the polymeric arms.^3,4^ Star polymers are the prototype of ultrasoft particles, with an interesting logarithmic dependence of the interaction potential on interparticle distance,^5^ which arises in the limit of very small core with respect to the length of the arms. The synthesis of multiarm stars is, however, quite involved^6^ and requires deep chemical expertise; this difficulty, together with the fact that the polymers used are typically dispersed in organic solvents, has partially hindered the availability of the samples and their wide spreading as a model system for experimental work. Furthermore, while examples of pH-responsive multiarm star polyelectrolytes are present,^7^ only a few recent examples of thermoresponsive star-like systems have been reported.^8–10^ These are however limited to small arm numbers and by a synthetic process that retains a high level of complexity. Thereby, the possibility to easily synthesize star-like particles dispersed in water, and even to provide them thermoresponsiveness, would be a major breakthrough in the field. On the other hand, microgels are soft colloidal particles consisting of a cross-linked polymer network, that are obtained by a much simpler synthesis. They are often dispersed in water and display responsive characteristics that are desirable for star polymers. Indeed, their polymeric nature endows them with the ability to respond to various external stimuli, such as light, pH, ionic strength, and temperature. The most widely investigated microgels are those based on poly­(*N*-isopropylacrylamide) (PNIPAM), a thermoresponsive polymer that exhibits a coil-to-globule transition at about 32 °C due to a change in solvent quality.^11^ This enables PNIPAM-based microgels to reversibly swell or shrink, modulating their effective volume fraction as a function of the temperature, through the so-called volume phase transition (VPT).^12^ PNIPAM-based microgels are typically synthesized via free radical precipitation polymerization.^13^ In this process, NIPAM monomers are dissolved in water along with cross-linking agents, most commonly *N*,*N*′-methylenebisacrylamide (BIS). Due to the differing reactivities of NIPAM monomers and BIS cross-linkers, the resulting microgels often adopt a core–shell morphology. This structure is characterized by a dense, cross-linker-rich core, surrounded by a looser, cross-linker-poor corona. The reference model to describe this characteristic structure is the well-established fuzzy-sphere model.^14^ NIPAM–BIS microgels are also extensively used as building blocks for more complex systems by using additional copolymers^15^ or decorating them with functional molecules or nanoparticles.^16,17^ The core–shell structure of these microgels is however still strongly different from that of a star polymer, resulting in markedly different properties.^18^ A few studies have explored variations in the synthesis conditions in order to obtain different internal structures of microgels, in particular toward the development of more homogeneous networks,^19–21^ while several recent works have addressed the case of ultralow-cross-linked microgels,^22,23^ in the absence of any external cross-linking agent, but relying on self-cross-linking mechanism of NIPAM. Even in the latter case, however, the density profile is significantly different from that of star polymers. To obtain a star-like architecture, it may be useful to use different types of cross-linkers. A pioneering study in this direction was put forward by the Hellweg group back in 2002,^24^ who reported the use of alternative cross-linking agents. In particular, these authors employed ethylene glycol dimethacrylate (EGDMA) instead of BIS, finding very high shrinking abilities of the particles even at large cross-linker content. They hypothesized that the fast reactivity of EGDMA, together with its tendency to self-bind, could be responsible for this peculiar behavior, however without providing detailed microscopic insights on the internal structure of the particles. A few additional studies on the topic are available in the literature on microgels not based on PNIPAM.^25,26^ More recently, in the context of copolymer microgels based on PNIPAM and poly­(ethylene glycol) (PEG) with EGDMA as cross-linker, it was found that the low temperature form factors could be be better described by a star polymer profile rather than by the standard fuzzy-sphere model.^27^

Inspired by the possibility to establish star-like behavior in microgels, in this work we provide an extensive experimental and computational study of PNIPAM–EGDMA microgels as a function of the EGDMA concentration, across the VPT. The microgels are synthesized in the presence of a fixed amount of surfactant, thereby not exceeding a few hundreds of nanometers in size. We thus complement dynamic light scattering (DLS) measurements with small-angle X-ray scattering (SAXS) and rationalize the experimental findings with monomer-resolved numerical simulations based on two different approaches: on the one hand, we employ *ad hoc*-designed star-like particles and on the other hand we build realistic microgels, based on the generalization of our previously established state-of-the-art method for standard PNIPAM-BIS microgels. We then put forward a suitable model, that relies on an appropriate combination of a star polymer colloid and a core-fuzzy-shell particle, hereafter named the “star-like fuzzy-sphere model”, that is able to describe the measured form factors for all cross-linker concentrations and temperatures. The analysis reveals a strong evidence of star-like morphology for PNIPAM–EGDMA microgels, particularly at low cross-linker concentration. Upon increasing the amount of EGDMA, the star-like character is retained despite the development of a distinct, cross-linker-rich core. Our work thus provides evidence of the realization of soft nanocolloids with star-like architecture, that are obtained with a standard precipitation polymerization synthesis in water. This peculiar internal structure is still combined with the thermoresponsive character of microgels, resulting in a very sharp VPT, which is preserved at all investigated EGDMA contents. These results hold high promise for applications as well as a fundamental model system, easily available by standard synthesis methods.

## Results and Discussion

### Swelling Behavior of PNIPAM–EGDMA Microgels

In Figure 1, we report the DLS characterization of the VPT of microgels synthesized with different contents of EGDMA, with molar percentages *C*_EGDMA_ between 0.5% and 10%. In particular, in Figure 1a, we report the normalized hydrodynamic radius *R*_H_(*T*)/*R*_H_(*T* = 20 °C) as a function of the temperature fitted through eq 1. For readability, we show the curves only for the samples with *C*_EGDMA_ of 1%, 5%, and 10% in Figure 1a, while all additional data are reported in Figure S1. The two key features of PNIPAM–EGDMA microgels are their high swelling ability and their capacity to maintain a sharp VPT even at high cross-linker concentrations. The swelling ability *S*_R_ = *R*_H_(*T* = 20 °C)/*R*_H_(*T* = 45 °C), namely, the ratio between the hydrodynamic radius in the swollen and the collapsed states, is shown in Figure 1b for all synthesized samples. For comparison, the dashed horizontal lines indicate the values of *S*_R_ for standard PNIPAM–BIS microgels. Notably, the swelling ratio of PNIPAM–EGDMA microgels with *C*_EGDMA_ = 10%, and even *C*_EGDMA_ = 15%,^24^ is comparable to that of standard microgels with markedly lower molar percentage of cross-linker *C*_BIS_ = 1%. In addition, the VPT remains very sharp even at high cross-linker concentrations, as shown directly from the swelling curves in Figure 1a and their fitted derivatives in Figure 1c. By fitting the data to eq 1, we quantify the transition sharpness via the parameter *s*, which is shown as a function of *C*_EGDMA_, in Figure 1d. By a comparison of the obtained values of *s* with those reported in the literature for PNIPAM–BIS microgels (*s* ∼ 0.9 for *c* = 1.4%^28^ and *s* ∼ 0.7 for *c* = 5%^29^), it is clear that the VPT for PNIPAM–EGDMA microgels occurs in a much narrower temperature range. In particular, the value of *s* for *C*_EGDMA_ = 5% is close to that of PNIPAM–BIS microgels with *C*_BIS_ = 1.4%,^28^ while the one for *C*_EGDMA_ = 10% is comparable with that for *C*_BIS_ = 5%.^29^ The main characteristics of PNIPAM–EGDMA microgels, as determined by DLS measurements, are summarized in Table S1.

**Figure 1:**
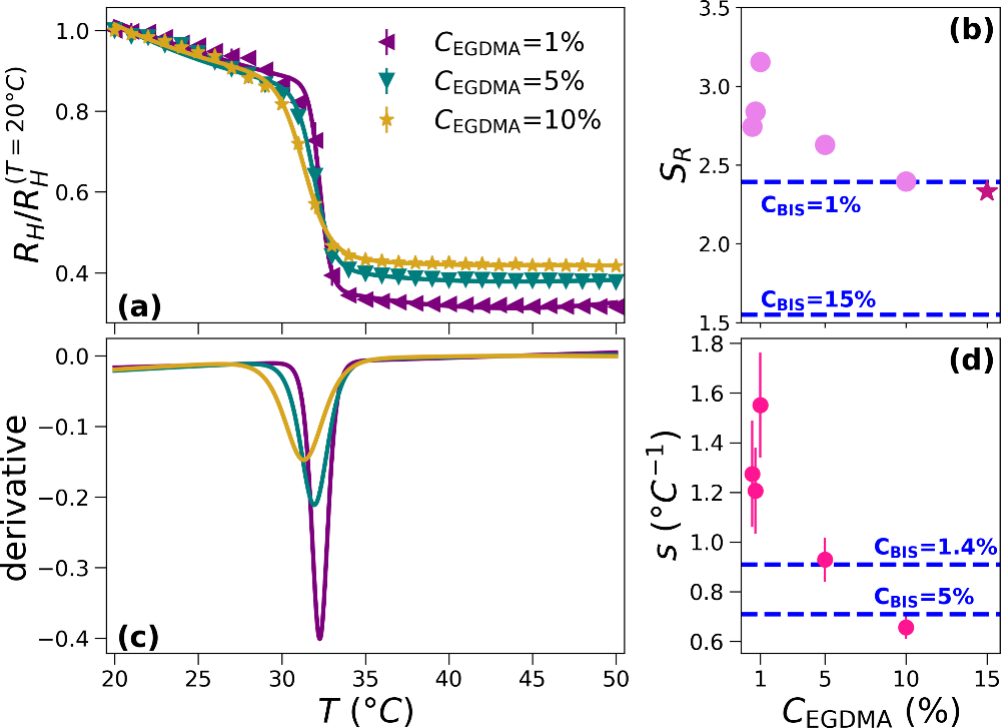
(a) Normalized hydrodynamic radius *R*_H_(*T*)/*R*_H_(*T* = 20 °C) of PNIPAM–EGDMA microgels with different EGDMA contents as a function of temperature *T* obtained by DLS. Solid lines are fits according to eq 1. (b) Swelling ratio *S*_R_ of the microgels as a function of the cross-linker concentration. The horizontal lines represent the corresponding values for microgels synthesized at *C*_BIS_ = 1% and *C*_BIS_ = 15%.^24^ The violet star represents *S*_R_ taken from the work of Kratz et al.^24^ (c) Derivative of the fits of the swelling curves reported in (a). (d) Sharpness parameter *s* extracted from the fits. The horizontal lines represent the corresponding value for microgels synthesized at *C*_BIS_ = 1.4%^28^ and *C*_BIS_ = 5%.^29^

### Evidence of a Star-Like Microscopic Structure of PNIPAM–EGDMA Microgels

To shed light on the microscopic structure, giving rise to the particular trend of the hydrodynamic radius as a function of the temperature, we performed SAXS measurements. Figure 2a shows the form factors for microgels with *C*_EGDMA_ = 1% as a function of the temperature. We can see that at low *T* the form factors display a peak-less shape that is maintained up to the VPT, where suddenly there is a change of structure, occurring between 31 and 32 °C. For higher temperatures *T* ≥ 35 °C, no further change of the form factors is detected in the whole investigated range. These results confirm the sharpness of the transition observed by DLS. Next, we focus on the form factors at two representative temperatures, below and above the VPT, shown in Figure 2b,c, respectively. Building on the hypothesis of Kratz and co-workers^24^ that there is an accumulation of cross-linkers in the core, we test a star-like morphology for our microgels. This is done in two ways: (i) we introduce a star-like fuzzy-sphere model to describe the experimental form factors, and (ii) we simulate star polymers with varying numbers of arms and core properties to compare with experimental data.

**Figure 2:**
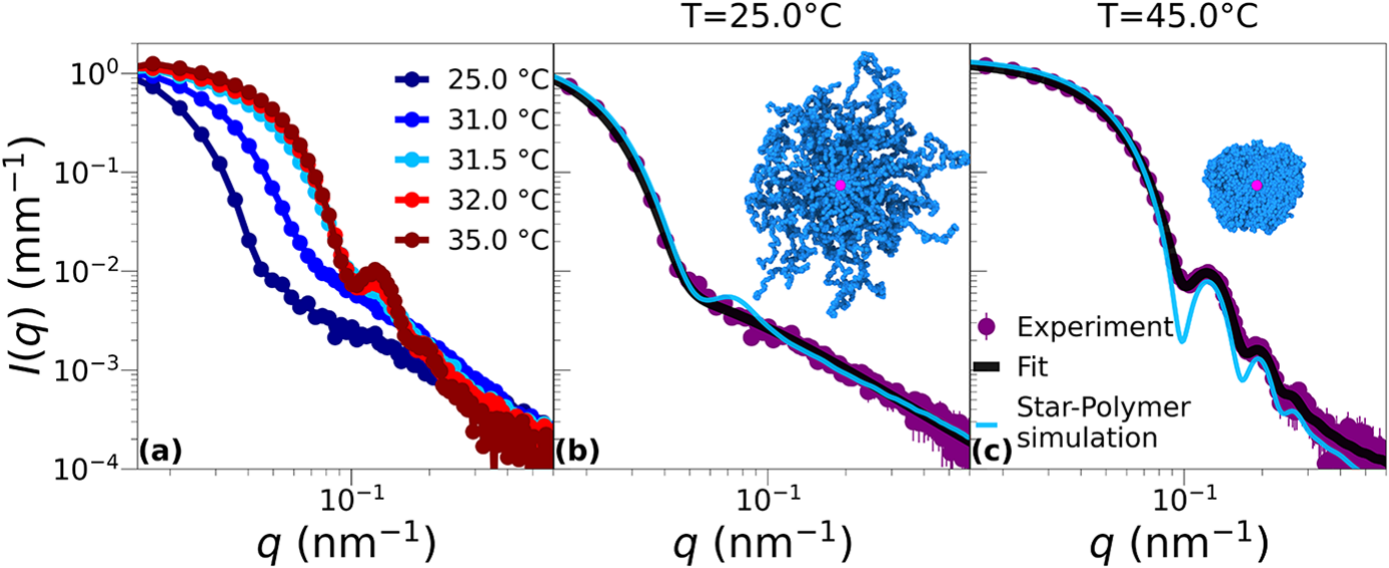
(a) SAXS scattered intensities *I*(*q*) for a sample with *C*_EGDMA_ = 1% measured at different temperatures. Note that for *T* ≥ 35 °C the data no longer change. (b and c) Measurements at *T* = 25 and 45 °C, respectively. Black lines are fits via the star-like fuzzy-sphere model (eq 3), while cyan lines are the form factors calculated for a simulated star polymer with *R*_c_ = 2.24σ, *f* = 80, and *N*_f_ = 200 whose snapshot and the corresponding slice at α = 0.0 (b) and 0.8 (c) are also shown. Here beads colored in blue represent monomers, while the core is the magenta central sphere.

We start by discussing the model, defined in eq 3 and described in detail in the Structural Model and Form Factor of Star Microgels section, that we developed in order to describe the peculiar structure of star-like microgels. In the model, the form factor is expressed as the sum of a term *P*_mgel_ (eq 4), which describes the overall structure of the particle, and a second term *P*_star_ (eq 6), already put forward by Dozier and co-workers,^30^ accounting for the short-range correlations inside the star corona. In particular, *P*_mgel_ is the form factor of a particle with a core complemented by a fuzzy shell that is needed in order to incorporate the mixed nature of star-like microgels, including both the fuzzy structure of microgels and a core region that can become more extended by increasing the amount of cross-linker. We find that eq 3 is able to quantitatively describe the experimental form factors of PNIPAM–EGDMA microgels with *C*_EGDMA_ = 1%, as shown in Figure 2b,c. Fit parameters for the two reported temperatures are summarized in Table 1. In particular, at *T* = 25 °C, the fit yields a rather small core radius, *R*_c_ = 5.6 ± 0.7 nm, shell thickness *t* = 48 ± 11 nm, and fuzziness σ_s_ = 47 ± 3 nm. The exponent μ is ≈0.66, in line with expectations for good solvent conditions (ν ≃ 0.588). The total radius of the microgels, *R*_t_ = *R*_c_ + *t* + 2σ_s_, is found to be consistent with the hydrodynamic radius *R*_H_(*T* = 25 °C) = 156 nm estimated by DLS and reported in Table S1. Indeed, we find *R*_t_(*T* = 25 °C) ≈ 148 nm. From the fit, we observe slight differences in scattering length density between the shell and solvent (Δρ^s0^) and between the core and shell (Δρ^cs^), consistent with the microgel being fully solvated. From the low-temperature form factors, it is not possible to estimate the polydispersity of the samples because the model reported in eq 3 is always able to fit the data independently of polydispersity. We also note that at low temperature an equally good fit is found also with the standard model for colloidal star polymers,^30^ as shown in Figure S2a. This indicates an internal structure that is fully compatible with that of a true star polymer. However, since to describe the behavior at high temperatures the star model is not sufficient, we rely on the star-like fuzzy-sphere model that works for all investigated conditions. Indeed, at high temperature, the microgel collapses and its shape becomes spherical. By fitting the form factor at *T* = 45 °C through eq 3, we find *R*_c_ = 38 ± 2 nm, *t* = 9 ± 1 nm, and σ_s_ = 3 ± 1 nm, giving a total radius *R*_t_ ≈ 53 nm, again in agreement with *R*_H_(*T* = 45 °C) = 51.3 nm (Table S1), while the value μ ≈ 1.7 signals the worsening of the solvent conditions. Given the peculiarity of the obtained structure, we test its robustness by using a different initiator in the synthesis. To this aim, we report in the Supporting Information the results for a PNIPAM–EGDMA microgel synthesized using the same fraction of EGDMA but employing ammonium persulfate (APS) rather than potassium persulfate (KPS) as the initiator. Figure S2b clearly shows that, also in the presence of APS, a star-like behavior is observed, and the data are again well described by the star-like fuzzy-sphere model.

**Table 1:** Best-Fit Parameters Derived from the Star-Like Fuzzy-Sphere Model (Equation 3) for Samples with *C*_EGDMA_ = 1% and 10%^a^

*C*_EGDMA_ (%)	*T* (°C)	*R*_c_ (nm)	*t* (nm)	σ_s_ (nm)	μ	ξ (nm)	Δρ^cs^ (nm^–2^)	Δρ^s0^ (nm^–2^)
1	25	5.6 ± 0.7	48 ± 11	47 ± 3	0.66 ± 0.03	21 ± 2	2.9 ± 0.2 × 10^–2^	1.1 ± 0.1 × 10^–2^
1	45	38 ± 2	9 ± 1	3 ± 1	1.74 ± 0.01	28 ± 1	2 ± 1 × 10^–2^	3.5 ± 0.5 × 10^–2^
10	25	28 ± 1	51 ± 1	25 ± 1	0.66 ± 0.05	20 ± 1	3.4 ± 0.1 × 10^–2^	1.2 ± 0.1 × 10^–3^
10	45	25 ± 1	28 ± 1	7 ± 1	1.60 ± 0.01	28 ± 1	8.4 ± 0.1 × 10^–3^	2.8 ± 0.1 × 10^–3^

a Δρ^cs^ and Δρ^s0^ represent the differences of the scattering length densities between the core and shell (ρ_c_ – ρ_s_) and between the shell and solvent (ρ_s_ – ρ_0_), respectively.

In order to validate the star-like architecture of the microgels at low *T*, we further compare the results with those calculated in the simulations of a star polymer, following the protocol described in the Experimental Methods and Numerical Methods sections. The *in silico* star that best reproduces the experimental microgel with *C*_EGDMA_ = 1%, also reported in Figure 2, is found to have a core radius *R*_c_ = 2.24 σ, with σ the size of a monomer in the simulations, as explained in the Numerical Methods section, arm number *f* = 80, and number of monomers per arm *N*_f_ = 200. These parameters fullfill the condition where the number of arms is able to fully cover the core, as in the standard blob description of a star.^5^ The model is capable of reproducing the deswelling behavior of the microgel by increasing temperature, using a value of the solvophobic parameter α = 0.8 in the collapsed state (see the Experimental Methods and Numerical Methods sections), which is the same α value that is normally employed to fit corresponding data for PNIPAM–BIS microgels.^31,32^ The snapshots of the star that best represents the microgel are shown as insets in Figure 2, denoting the presence of a tiny finite core, which is responsible for the small peak present in the numerical form factor at α = 0.0.

Next, we move on to examine the experimental form factors of microgels with *C*_EGDMA_ = 10%. These are reported in Figure 3a at different temperatures. Here a more complex structural change is observed at low *T*, as compared to Figure 2, but still the microgel is found to sharply collapse around *T* = 32 °C, and for *T* ≥ 35 °C its structure does not change any longer. We then focus on the SAXS data acquired at low and high temperatures, shown in parts b and c of Figure 3, respectively, together with the fits performed using the star-like fuzzy-sphere model of eq 3, which is found also in this case to quantitatively describe the measured data at both temperatures. The resulting fit parameters are reported in Table 1, showing that the size of the inner core *R*_c_ remains roughly constant upon varying the temperature. In particular, we find *R*_c_(*T* = 25 °C) = 28 ± 1 nm and *R*_c_(*T* = 45 °C) = 25 ± 1 nm. This is the signature of the presence of a stiff central region with a large density of cross-linkers, which does not strongly respond to temperature changes. Instead, a significant decrease is observed for both the shell thickness and the fuzziness, by increasing *T*. The total size of the microgels is found to be consistent at both temperatures with the hydrodynamic radius determined by DLS *R*_H_(*T* = 25 °C) = 141.7 nm and *R*_H_(*T* = 45 °C) = 62.5 nm, as reported in Table S1. Indeed, we find *R*_t_(*T* = 25 °C) ≈ 129 nm and *R*_t_(*T* = 45 °C) ≈ 67 nm. From the fit, we obtain a polydispersity of 7% in *R*_c_ at low temperature, while at high *T*, the model fits the data very well without including polydispersity. As done in the case *C*_EGDMA_ = 1%, we tried to compare the experimental form factors with those calculated in the simulations of a star polymer (Figure S3). In this case, because EGDMA forms a larger central core, the simulated star polymer fails to describe the experimental results, as detailed in the Supporting Information. Hence, we can only capture the qualitative behavior at low *T* but not at high *T*. Altogether these results suggest that, as *C*_EGDMA_ increases, the resulting structure at low temperatures differs from that of a simple star polymer, becoming more similar to a core-fuzzy shell. This observation points to an internal structure that combines features of a star-like object with a finite central core and those of a microgel exhibiting a fuzzy density profile, thereby establishing the star-like fuzzy-sphere model of eq 3 as the most appropriate to correctly capture PNIPAM–EGDMA microgels, independently of the amount of EGDMA.

**Figure 3:**
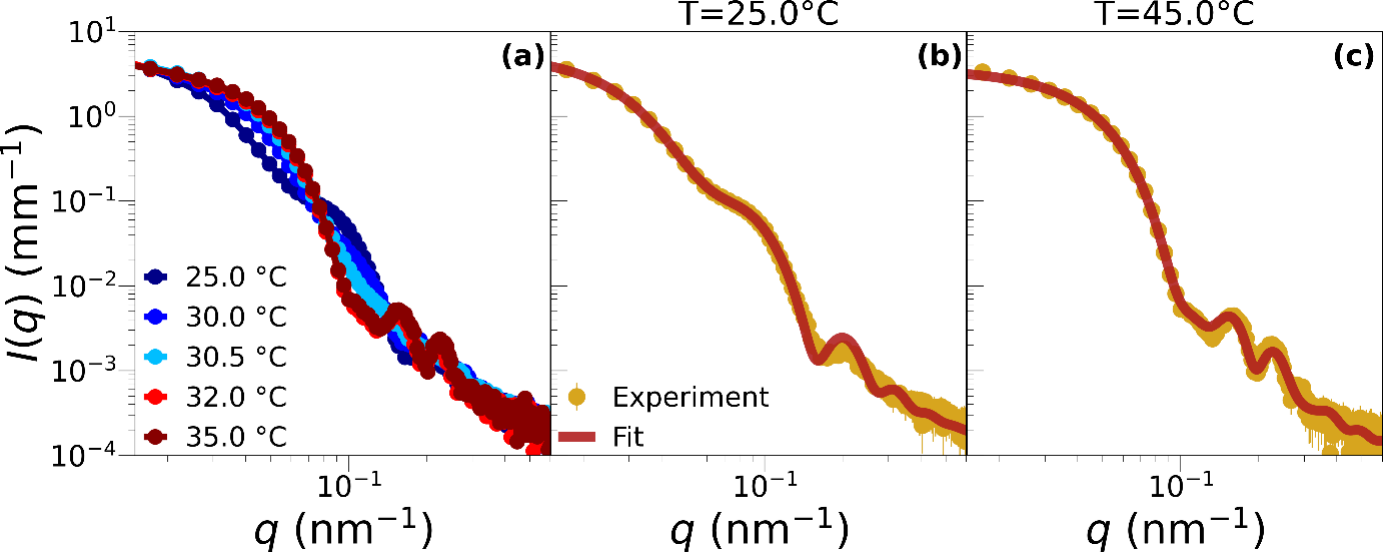
(a) SAXS scattered intensities *I*(*q*) for a sample with *C*_EGDMA_ = 10% measured at different temperatures. Note that, for *T* ≥ 35 °C, the data no longer change. Panels (b) and (c) focus on the measurements at *T* = 25 and 45 °C. Brown solid lines represent fits in which the form factor *P*(*q*) is described by the star-like fuzzy-sphere model presented in eq 3.

### Monomer-Resolved Simulations of Star-Like Microgels

To get microscopic insights on this behavior, we build on our previous works on PNIPAM–BIS microgels and develop a monomer-resolved microgel model, able to capture the PNIPAM–EGDMA architecture. As described in the Experimental Methods and Numerical Methods sections, we generalize our recipe put forward in refs 31 and 32 to take into account the extremely fast polymerization kinetics of EGDMA and the tendency of the cross-linking molecules to bind among themselves. In this way, we are able to reproduce the full form factor experimental behavior, upon varying *C*_EGDMA_ and temperature, with a consistent set of parameters that uses the same nominal *C*_EGDMA_ as in experiments.

The comparison between the calculated form factors for the *in silico* PNIPAM–EGDMA microgels and experiments is reported in Figure 4, again for the two representative temperatures *T* = 25 and 45 °C, respectively in panels a and b, for both *C*_EGDMA_ = 1% and *C*_EGDMA_ = 10%. The agreement between simulations and experiments is quite accurate. This is obtained by simply rescaling the simulation data onto the experimental ones, as done in previous works,^31^ using the monomer size σ = 0.48 and 0.43 nm, respectively, for the two microgels. These values are then maintained at all temperatures. We find that the high-*T* data are well-captured using an effective temperature α = 0.8, consistently for the star polymer model in Figure 2 and for PNIPAM–BIS microgels.^31,32^ These results highlight the robustness of our *in silico* microgel preparation, making it able to reproduce microgels with very diverse architectures. Furthermore, the availability of a microscopic model enables us to look at the internal structure of the PNIPAM–EGDMA microgels in real space. We thus report the radial density profiles of the microgels in parts c and d of Figure 4 respectively for low and high temperatures. Together with the full profiles, we also isolate the density profile of the cross-linkers, also reported in Figure 4c,d. They do indicate that the cross-linkers are clearly localized in a tiny region of space, the particles core, whose size changes very little with increasing temperature. These findings indicate that the chains departing from the core can thus be considered as true isolated arms, as in star polymers. This is confirmed by the snapshots of the microgels, and the corresponding slices to highlight the cross-linker-rich core, reported in Figure 5. It is clear that for *C*_EGDMA_ = 1% the core is entirely made of cross-linkers and no cross-linkers are outside of it. However, it is also evident that the arms are widely polydisperse in length and we also find that sometimes they even connect from one point to another on the core. Estimating the core radius as *R*_c_ ∼ 5.7 σ from a Gaussian fit of the cross-linker density profile, we obtain that it is quite small in comparison to the total microgel size and that its inner packing fraction is ≈0.28. We then count the number of arms departing from the core, getting approximately *f* ∼ 200 for *C*_EGDMA_ = 1% and a surface coverage fraction^1^ γ = *f*/16*r*_c_^2^ ∼ 0.38. Although the core coverage is only partial, different from the case of an ideal star polymer model, our microgel still shows a density profile that is quite compatible with the star polymer predictions at large distances (see the Experimental Methods and Numerical Methods sections and eq 16), as indicated by the fits also reported in Figure 4c. This behavior is found to be valid also for the more cross-linked microgel. Clearly, the density profiles of the microgels with both concentrations of EGDMA are very different from the corresponding ones obtained for PNIPAM–BIS microgels, reported in the inset of Figure 4c, indicating a very different structure also at high EGDMA content. In the latter case, the core becomes much more extended, exceeding the scaling behavior with ∼*C*_EGDMA_^1/3^, since we observe from the snapshots in Figure 5 that a significant fraction of monomers also remains trapped in the core. Therefore, for *C*_EGDMA_ = 10%, it is not obvious to identify a clear star structure and also to count the arms departing from the core. We thus believe that the additional monomers belonging to the core give rise to the fuzziness of the particles, that is needed to describe the experimental form factors, included in the star-like fuzzy-sphere model. At high temperature, the simulations fully capture the evolution of the peak positions, including the depletion of the first peak observed for *C*_EGDMA_ = 10%, that is quite reminiscent of that observed in core–shell microgels where the core is a solid silica core,^33^ again reinforcing the choice of our star-like fuzzy-sphere model. We note that the peaks are less pronounced in the simulations, probably due to finite size effects, as increasing the number of monomers is known to increase the high-*q* oscillations.^31^ We also report in Figure S4 the comparison between the density profiles of *C*_EGDMA_ = 1% star microgel and *C*_EGDMA_ = 1% star polymer, to show that they are quite similar to each other and also compatible with eq 16. Altogether these simulations show that, even if our microgels are built from the self-assembly of monomers and cross-linkers mixed in the same stoichiometric ratio as in experiments and not as designed stars, we do recover a star-like structure that is mixed with microgel features for these fascinating soft particles.

**Figure 4:**
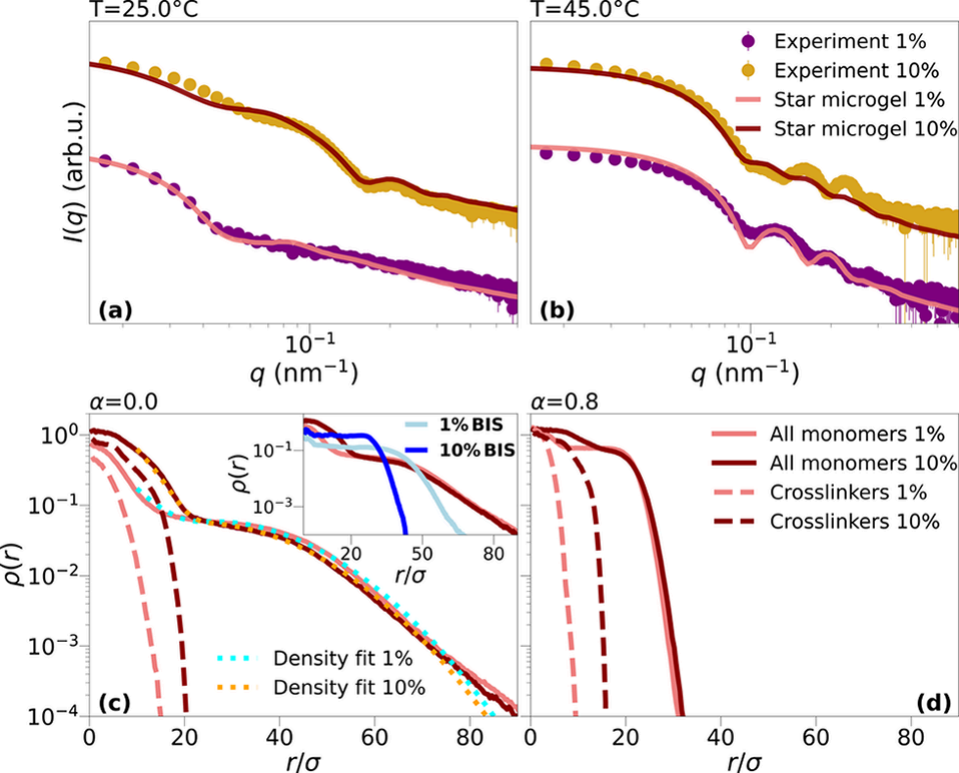
SAXS scattered intensities *I*(*q*) for PNIPAM–EGDMA microgels with *C*_EGDMA_ = 1% and 10% at *T* = 25 °C (a) and 45 °C (b). The curves are vertically shifted for clarity. Solid lines represent the form factors of simulated star microgels at α = 0.0 (a) and 0.8 (b). Numerical curves are superimposed onto experimental ones by rescaling for the monomer size σ = 0.48 and 0.43 nm, respectively for *C*_EGDMA_ = 1% and 10%. In (c) and (d), we show the density profiles obtained from simulations of star-microgels at α = 0.0 and 0.8, respectively, as a function of the distance from the center of mass, rescaled by the monomer size. Density profiles at α = 0.0 are well-fitted by eq 16, representative of star behavior, shown as dotted lines. Full lines correspond to the whole microgel profile, while dashed lines are the profiles of the cross-linkers only. The inset in (c) shows the low-*T* comparison between the density profiles of PNIPAM–BIS and PNIPAM–EGDMA microgels for both values of cross-linker concentrations, 1% and 10%.

**Figure 5:**
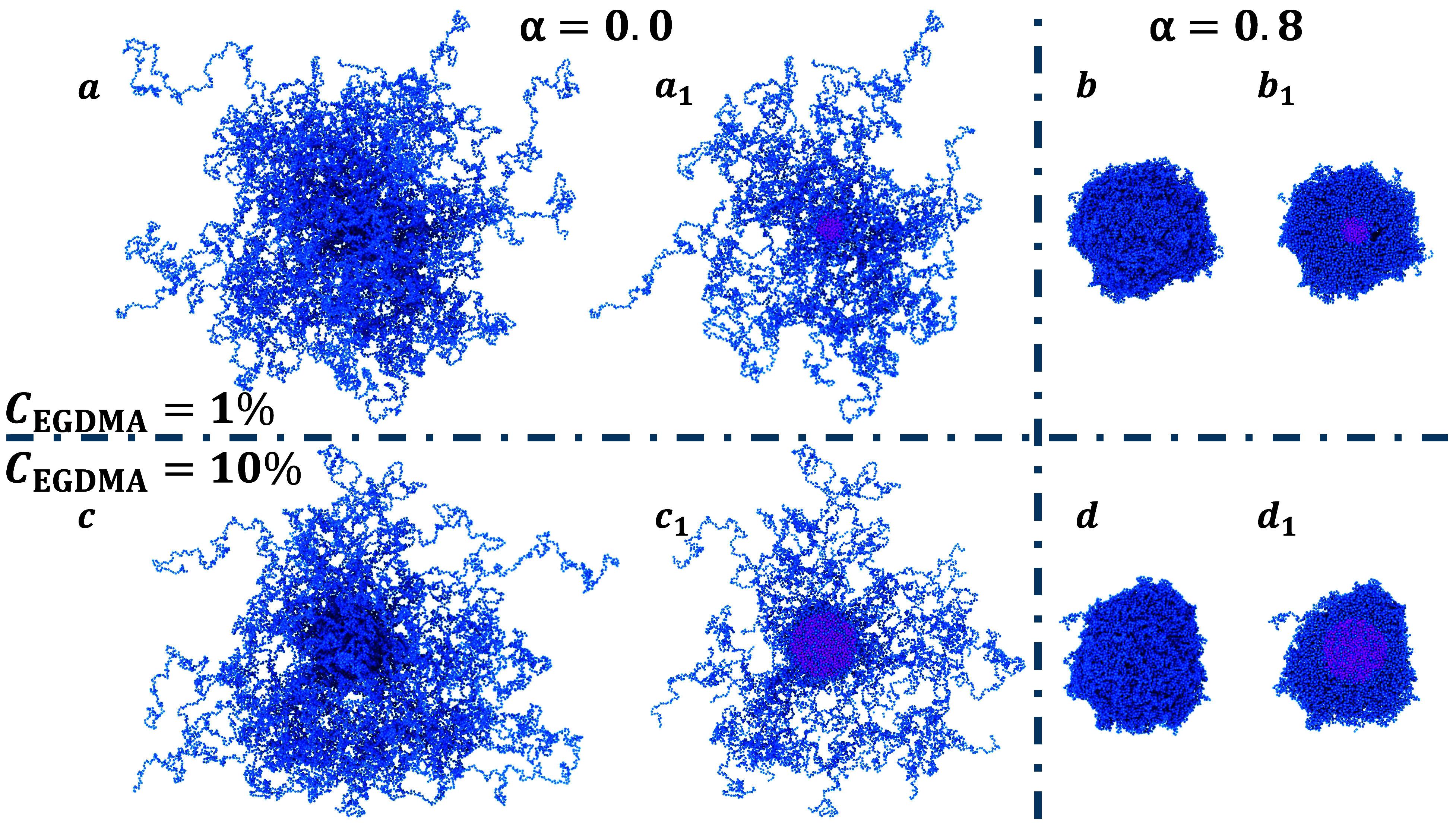
Snapshots of simulated PNIPAM–EGDMA star-like microgels with *C*_EGDMA_ = 1% and 10% at α = 0.0 (a and c) and 0.8 (b and d). The corresponding slice is shown next to each snapshot to highlight the cross-linkers, colored in magenta, for *C*_EGDMA_ = 1% (a_1_ and b_1_) and for *C*_EGDMA_ = 10% (c_1_ and d_1_). Note that the core is made by cross-linkers as well as a significant fraction of monomers for the *C*_EGDMA_ = 10% case.

To further analyze the star-like microgels synthesized with EGDMA, we now proceed with a detailed comparison of the experimental form factors with those calculated with the simulated microgels in the full temperature range. In this way, we can establish a mapping between the temperature in degrees Celsius and the solvophobic parameter α, similarly to what was found for PNIPAM–BIS microgels.^31^ In Figure 6a, we compare numerical and experimental *P*(*q*) for *C*_EGDMA_ = 1% for several values of temperature and the corresponding solvophobic parameter α. The resulting mapping between α and *T*, also found to be valid for *C*_EGDMA_ = 10% microgels, is reported in the inset of Figure 6b, together with the same mapping done for standard PNIPAM–BIS microgels by Ninarello et al.^31^ Curiously, we find that while the VPT temperature remains the same, the two types of microgels explore the temperature range with a rather different effective solvophobic potential. This is due to the absence of cross-linkers in the outer region of the star-like microgels, which makes the transition from swollen to collapsed rather sharp, in comparison to standard microgels, where the presence of cross-linkers in the corona gives rise to a more cooperative transition. The direct comparison between the swelling curves from experiments and simulations for PNIPAM–EGDMA microgels is finally shown in Figure 6b, where the normalized hydrodynamic radii measured from DLS are reported together with the normalized gyration radii from simulations. Although the two quantities are not strictly the same, the comparison indicates a rather good agreement. Additionally, in Figure S5, we show the comparison between the normalized radius of gyration of *in silico* PNIPAM–BIS and PNIPAM–EGDMA microgels to note once again that with the model introduced in the simulations we are able to obtain star-like microgels that have a high swelling capacity and a very sharp VPT.

**Figure 6:**
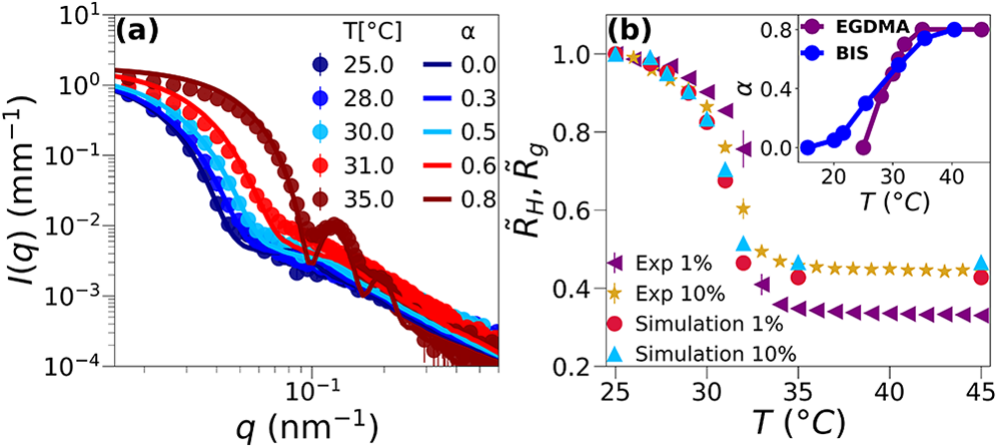
(a) Comparison between experimental (full symbols) and numerical form factors (solid lines) of PNIPAM–EGDMA microgels with *C*_EGDMA_ = 1%. The numerical form factors are rescaled along the *x* axis by the same factor σ = 0.48 nm at all temperatures. (b) Comparison between the normalized hydrodynamic radius *R̃*_H_ = *R*_H_/*R*_H_(*T* = 25 °C) measured with DLS and normalized radius of gyration *R̃*_g_ = *R*_g_/*R*_g_(α = 0.0) calculated from simulations as a function of the temperature. The mapping between the solvophobic parameter α and *T* is shown in the inset and compared with that of PNIPAM–BIS microgels.^31^

## Conclusions

In this work, we presented an extensive characterization through experiments and simulations of an emerging class of soft nanocolloids: PNIPAM–EGDMA microgels. Although they were first reported in 2002,^24^ and sometimes EGDMA is used to replace BIS as the cross-linker in studies of copolymerized microgels,^27,34–37^ no dedicated study has yet addressed their peculiar inner structure. Building on the fascinating hypothesis that EGDMA could concentrate in the middle of the microgels due to its fast reactivity, we have measured their form factors with high-brilliance SAXS at the ESRF and compared them with state-of-the-art simulations to finally reveal a quite complex architecture.

Indeed, we demonstrate that PNIPAM–EGDMA microgels are structurally very similar to ideal star polymers for low cross-linker concentration. This opens the possibility to investigate these very intriguing systems, largely studied in the past theoretically and also experimentally, whose spreading in the last years has been hindered due to their difficult synthesis. The use of a simple polymerization reaction is indeed a major breakthrough to their exploitation, particularly to investigate dynamical behavior and rheology of dense suspensions.^38,39^ At present, we have shown that the number of arms is set by the synthesis in the order of ∼10^2^, but we expect that modifications of the simple batch approach, e.g., semibatch, could be employed to vary the number of arms and to tune the properties of the stars. Furthermore, upon increasing EGDMA concentration, we have observed that the microgels acquire a larger core, but still maintain arms that are basically non-cross-linked to each other, which would be also interesting to compare to polymer-grafted nanoparticles.^40^ Here, the peculiarity of the present soft particles is that the core is not solid, and maintains some degree of thermoresponsiveness and of fuzziness, as shown by our form factor description. This hybrid nature makes star-like microgels very appealing for applications in different fields, as they bridge properties of particles that were classified as distinct classes in the soft matter catalogue, but here they can be tuned simply changing the EGDMA content and perhaps introducing additional parameters to the synthesis, e.g., copolymers. It will also be important to classify the softness of star-like microgels in the context of existing ones.^38,41^

To characterize the unusual structure of star-like microgels we have introduced a model which combines star polymer and microgel features, able to describe the form factors of the microgels at all temperatures and EGDMA concentrations. This model also reveals the strong star-like character of the microgels at low cross-linker concentration, that are very similar to true star polymers, as also demonstrated by numerical simulations. In addition, we have shown that our monomer-resolved PNIPAM–EGDMA microgel model is able to describe the experimental samples with a consistent set of parameters, using very simple and intuitive modifications of our previously established assembly of PNIPAM–BIS microgels. Specifically, we modified the range and the strength of the force acting on the cross-linkers during assembly in order to mimic the faster reactivity of EGDMA compared to BIS, and we then varied the range of this force consistently with the concentration of EGDMA. These findings, on the one hand, reinforce the potential of the *in silico* synthesis, which can be adapted to describe microgels of virtually any structure, and, on the other hand, pave the way for further investigations into star-like microgels in different conditions, e.g., at interfaces^42,43^ or in dense suspensions,^44,45^ as already done for PNIPAM–BIS microgels. In addition, the theoretical characterization of microgel–microgel interactions will be important, particularly for comparison with the logarithmic effective potential expected^1^ for star polymers. Potentially, our investigation will open interesting perspectives in the study of soft nanocolloids, addressing the physical interplay between elastic (microgels) and ultrasoft (stars) interactions.

Finally, from the point of view of applications, it is important to stress that PNIPAM–EGDMA microgels provide star-like soft particles with inherent thermoresponsivity, a feature giving rise to the occurrence of a VPT that is much sharper than that in standard PNIPAM–BIS microgels. This behavior, already observed in the pioneering work of Hellweg and co-workers,^24^ is now rationalized by the star-like architecture, which allows a large freedom to the arms, thus being able to experience more suddenly the increased hydrophobic interactions with the solvent with respect to a cross-linked corona. The obtained sharp transition can be of course very appealing for all those applications requiring a sudden volume change of the microgels, e.g., sensing or drug delivery.

## Experimental Methods

### Chemicals

The *N*-isopropylacrylamide monomer (NIPAM; Sigma-Aldrich) was recrystallized from hexane and methanol and then dried under reduced pressure (0.01 mmHg) at room temperature. Ethylene glycol dimethacrylate (EGDMA), sodium dodecyl sulfate (SDS) with 98% purity, potassium persulfate (KPS), and ammonium persulfate (APS) (all purchased from Sigma-Aldrich) were used as received.

### Synthesis of the Microgels

Microgels with different molar percentages of cross-linker EGDMA (from 0.5% to 10%) were prepared under precipitation method conditions. Specifically, NIPAM monomers, cross-linker EGDMA, and the surfactant SDS were dissolved in 26.5 mL of Milli-Q water and placed in a 50 mL two-necked reactor, equipped with a condenser and a magnetic stirrer. The reactor is immersed in an oil thermal bath whose temperature is controlled through a heating plate. The solution was purged under a nitrogen stream for 1 h at room temperature. Subsequently, the temperature was raised to 70 °C, and then polymerization was initiated by a controlled addition of KPS (11.6 mg dissolved in 1.2 mL of deoxygenated water) at a rate of 1 mL/min. The reaction was carried out at 70 °C for 5 h. The resulting PNIPAM microgels were purified by dialysis in a cellulose membrane (MWCO: 6–8 kDa) against ultrapure water for 2 weeks, with water changes twice daily. The microgel solutions were then freeze-dried and stored in the dark at 4 °C. The exact amounts of reagents used in the different syntheses are provided in Table 2.

**Table 2:** Quantities of Reagents Used for the Syntheses of Microgels

*C*_EGDMA_ (%)	EGDMA (μL)	NIPAM (mg)	SDS (mg)
0.5	4.16	497.2	12.7
0.7	5.83	496.2	12.7
1	8.30	494.7	12.7
5	41.60	474.7	12.7
10	83.30	449.7	12.7

### Dynamic Light Scattering (DLS)

The hydrodynamic radius, *R*_H_, was measured as a function of the temperature using DLS. Measurements were performed with a NanoZetaSizer apparatus (Malvern Instruments Ltd.) equipped with a He–Ne laser (5 mW power, 633 nm wavelength) that collects light at an angle of 173°. Measurements were carried out diluting the samples to a concentration of *C* = 0.01 wt % in Milli-Q water in the temperature range between 20 and 50 °C. After each temperature change, we equilibrated the sample for 5 min. The hydrodynamic radius *R*_H_ and polydispersity index were determined by cumulant analysis.^46^

To describe the swelling behavior, we fit the hydrodynamic radius versus temperature with the function^28^

1 $$R_{H}\left(T\right)=R_{0}-\Delta R\tanh\left[s\left(T-T_{c}\right)\right]+A\left(T-T_{c}\right)+A_{1}{\left(T-T_{c}\right)}^{2}+A_{2}{\left(T-T_{c}\right)}^{3}$$

where *R*_0_ is the radius of the microgel at the VPT, *T*_c_ is the VPT temperature, Δ*R* is the amplitude of the VPT, and the parameter *s* quantifies its sharpness. The function includes a third-order polynomial inserted to describe well the trend of the hydrodynamic radius over the entire temperature range even far from *T*_c_. To characterize the VPT, we also evaluate the swelling ratio *S*_R_ = *R*_H_(*T* = 20 °C)/*R*_H_(*T* = 45 °C).

### Small Angle X-ray Scattering (SAXS)

SAXS experiments were performed at the ID02 beamline of the ESRF.^47^ Samples were measured at low concentration of 0.1 wt %, which ensures direct measurement of the microgel form factor. Indeed, the SAXS scattered intensities can be expressed as

2 $$I\left(q\right)=\phi V{\left(\Delta \rho \right)}^{2}P\left(q\right)\;S\left(q\right)$$

where ϕ is the particle volume fraction, *V* the particle volume, Δρ the scattering length density difference between the microgels and solvent, *P*(*q*) the particle form factor, and *S*(*q*) the structure factor. Because we performed measurements in diluted conditions, the contribution of the structure factor can be neglected; thus, the measured curves are directly linked to the form factor through a multiplicative prefactor. For measurements, samples were placed in quartz capillaries (2 mm diameter). We acquired SAXS curves using a Eiger2X 4 M pixel detector, at temperatures selected between 25 and 45 °C using a Peltier stage; after each temperature change, samples were equilibrated for 5 min before measurements. The sample–detector distance was set to 5 m in order to achieve a *q* range of 1 × 10^–2^ nm^–1^ ≤ *q* ≤ 1 nm^–1^ where *q* is defined as *q* = (4π/λ) sin θ, 2θ is the scattering angle, and λ is the wavelength of radiation. The exposure time for acquisitions was set to 0.5 s and 10 scattering patterns were acquired for each temperature. Scattering patterns of a capillary filled with water were recorded for background subtraction. The processing and averaging of the scattering patterns were performed by the software *SAXSutilities2*.^48^ When averaging, any scattering curve not perfectly superimposed with the overall set acquired, due to possible residual equilibration or other experimental perturbations, was discarded. Curve fitting was carried out using the software *SasView*,^49^ employing the model for particle form factors introduced in the following section.

### Structural Model and Form Factor of Star Microgels

We introduce a model that is able to describe the internal structure of star-like microgel particles and we derive an analytical expression of their form factor. The particles are composed by a highly cross-linked core and an external corona made primarily of free arms. To represent the scattering intensity of such particles, our model features two distinct regimes, taking place at different length scales. Therefore, following the standard assumptions adopted for deriving the form factors of colloidal star polymers and microgel particles,^30,35^ we directly sum the corresponding scattering intensities and neglect any possible interference between them, as

3 $$P\left(q\right)=A_{1}P_{\mathrm{mgel}}\left(q\right)+A_{2}P_{\mathrm{star}}\left(q\right)$$

Here, the first term, *P*_mgel_, describes the overall structure of the particle, whereas the second one, *P*_star_, accounts for the short-range correlations inside the star corona. The two contributions are weighted by the constants *A*_1_ and *A*_2_.

For *P*_mgel_(*q*), that is dominant at low *q*, we use a “core-fuzzy shell” model, consisting of a spherical core with radius *R*_c_ and scattering length density ρ_c_, and a fuzzy shell with external radius *R*_s_, fuzziness parameter σ_s_, and scattering length density ρ_s_. This allows one to account for the fuzzy edge of the star corona, in analogy to ref 14, and for its different contrast with respect to the cross-linked core. The analytical expression of *P*_mgel_(*q*), sketched in Figure 7, is

$$P_{\mathrm{mgel}}\left(q\right)=\frac{1}{V_{\mathrm{tot}}}\times {\left[\Delta \rho^{cs}A_{s}\left(q,R_{c}\right)+\Delta \rho^{s0}A_{s}\left(q,R_{s}\right)\;\exp\left(-\frac{1}{2}{\sigma_{s}}^{2}q^{2}\right)\right]}^{2}$$ 4

where ρ_0_ is the scattering length density of the solvent, Δρ^cs^= (ρ_c_ – ρ_s_) is the difference between the scattering length density of the core and the shell, Δρ^s0^= (ρ_s_ – ρ_0_) is the one between the shell and the solvent, $V_{\mathrm{tot}}=\frac{4}{3}\pi {R_{t}}^{3}$ is the total volume of the particle (*R*_t_ ≈ *R*_s_ + 2σ_s_ ^14^), and *A*_s_(*q*,*R*) represents the scattering amplitude of a homogeneous sphere of radius *R*, written as

5 $$A_{s}\left(q,R\right)=3V\frac{\sin\left(qR\right)-qR\cos\left(qR\right)}{{\left(qR\right)}^{3}}$$

where $V=\frac{4}{3}\pi R^{3}$ . Within this model, we thus define the thickness of the shell *t* = *R*_s_ – *R*_c_. For *P*_star_(*q*), we use the expression provided by Dozier et al.:^30^

6 $$P_{\mathrm{star}}\left(q\right)=\frac{4\pi }{q\xi }\frac{\sin\left[\mu \arctan\left(q\xi \right)\right]}{{\left(1+q^{2}\xi^{2}\right)}^{\mu /2}}\Gamma \left(\frac{\mu }{2}\right)$$

where μ = (1/ν) – 1, with ν the Flory solvency parameter, Γ­(μ/2) is the gamma function with argument μ/2 and ξ is the maximum size of the polymer chain blobs, the spherical regions within which each arm of the star exhibits single-chain behavior,^50^ representing the characteristic length scale at which the granular structure becomes relevant. Hence, this term is dominant at large *q*. Because almost all of the scattering intensity comes from the largest blobs, namely, the outermost ones,^30^ there is no need to adjust this term because the fact that the chains stem from the surface of the core rather than from the particle center has no significant effect.

**Figure 7:**
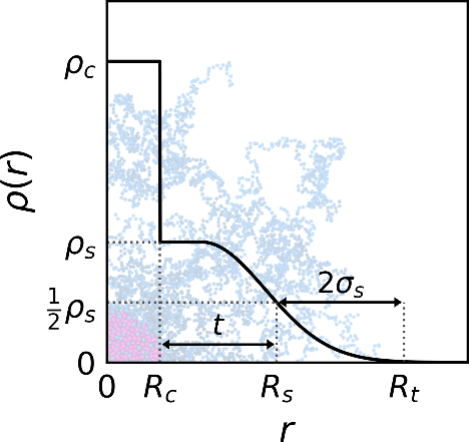
Sketch of the radial density profile of the “core-fuzzy shell” model for star-like microgels.

It is worth noting that in the model for colloidal star polymers of Dozier et al.^30^ the form factor is described by the sum of *P*_star_(*q*) and of a Guinier term *P*_G_(*q*), which only accounts for the gyration radius *R*_g_ of the particle:

7 $$P_{G}\left(q\right)\propto \exp\left(-\frac{1}{3}q^{2}{R_{g}}^{2}\right)$$

which has been replaced in the present analysis by the core-fuzzy shell term *P*_mgel_. However, when we fit the data with the simple star model in Figure S2a, we refer to the original Dozier model, including the Guinier term.

## Numerical Methods

### Design of Star Polymers

We simulate star polymers with a central core surrounded by *f* arms, each made on *N*_f_ identical beads of diameter σ. We adopt two strategies: in the first one, the core is composed of a single particle of radius *R*_c_ and, in the second one, which is more suitable to comparing to experiments in the case of a large core at high EGDMA concentration, we make a core that is formed by several beads, again of radius *R*_c_, to mimic the microgel situation, where the core is not a single particle. In this second approach, the core is designed by uniformly generating monomer positions within a sphere using a random sequential addition algorithm that maintains a minimum distance to prevent overlaps. A KDTree is then constructed to efficiently locate nearby monomers, forming bonds based on a cutoff distance. Each monomer is limited to a maximum of four bonds; any excess connections are pruned by removing the longest bond iteratively. Each arm is then anchored by selecting a core atom as the attachment point, with the constraint that each core atom can serve as a single attachment point. The radial vector from the origin to the chosen core atom defines the chain’s orientation. The first monomer is placed at a fixed distance from the core, and subsequent monomers are sequentially positioned along the same radial direction at intervals equal to the bond length, avoiding particle overlaps. Once the starting configuration is obtained, it is then run with the *LAMMPS* simulation software, as described in the following also for microgels.

### *In Silico* Synthesis of Microgels

To make PNIPAM–EGDMA microgels *in silico* we exploit our previous assembly method developed for standard (PNIPAM–BIS) microgels.^31,51^ In particular, we start using a binary mixture of *N* = 42000 patchy particles of mass *m* and diameter σ, respectively the units of mass and length, within a spherical cavity of radius *Z*. Monomers are represented as divalent particles, while cross-linkers have a valence of four. The molar ratio of cross-linkers *C* = *N*_c_/*N* used in experiments is the same as the experimental one, varying from 1% to 10%.

To reproduce the experimental fuzzy-sphere structure of standard microgels, an additional force is added on the cross-linkers, which mimics their faster reactivity with respect to NIPAM monomers. This radial force was optimized for BIS cross-linkers in ref 31 to take the following form:

8 $${\mathrm{f⃗}}_{d}=\{\begin{array}{ll} -kr\mathrm{r̂}, & \mathrm{if}0<r\leq D\\ -g\mathrm{r̂}, & \mathrm{if}D<r<Z \end{array}$$

where *ȓ* is a unit vector pointing outward from the center of the cavity, *D* = *Z*/2 is an intermediate length within which cross-linkers are mostly confined, and *k*_BIS_ = 4.5 × 10^–5^ϵ/σ^2^ and *g*_BIS_ = 0.008ϵ/σ are two phenomenological constants, where σ and ϵ are units of length and energy, respectively. The monomer number density ρ_mon_ is constant to the value ρ_mon_ = 3*N*/(4π*Z* ^3^) ≃ 0.08σ^–3^ whose value was validated against experiments at different cross-linker concentrations.^32^

This methodology needs to be modified in order to take into account the much larger reactivity of EGDMA. In particular, we varied the parameters of radial force acting on the cross-linkers, while maintaining it still of the same form of eq 8. We find that, to reproduce PNIPAM–EGDMA microgels, we need to use values of the constants in the force that are 2 orders of magnitude larger than those employed for standard PNIPAM–BIS microgels, i.e.

9 $$k_{\mathrm{EGDMA}}=4.5\times 10^{-3}\epsilon /\sigma^{2}$$

10 $$g_{\mathrm{EGDMA}}=0.8\epsilon /\sigma$$

In addition, we also find that it is crucial to allow cross-linkers to bind among themselves as well as with NIPAM, something that could be neglected in the case of standard microgels but is compulsory here in order to be able to assemble the microgels. Finally, we need to use a much more reduced size of the confining length *D*, to mimic the small size of the core made up of cross-linkers, which needs to increase with the cross-linker concentration *C* as ∼*C* ^1/3^. We thus determine *D* = 2σ and 4.3σ for *C* = 1% and 10%, respectively. This recipe is thus, in principle, applicable to any microgel of this kind by simply rescaling *D* with *C* and *N*, both with a spherical growth law with exponent 1/3. The monomer number density is always kept fixed to ρ_mon_. Once the force on the cross-linkers is set, assembly simulations are conducted at low temperature employing the *oxDNA* package^52^ with the additional swap protocol^53^ to facilitate network formation as in previous works.^51^ We wait until ≫99% of the possible bonds is formed, and then we retain the largest cluster only. Importantly, we find that all input cross-linkers react and go to the core of the assembled structure, but there is quite a waste of the remaining monomers during the *in silico* synthesis, so that we end up with the final number of beads in the microgel reducing to ∼30000 and ∼35000 for *C* = 1% and 10%, respectively.

### Interaction Potentials

Once the star polymer is built or the microgel is assembled, the interactions between all particles (also called beads in the following), including monomers, cross-linkers, and also the core of the stars, are modeled with the bead–spring model,^54^ which is able to capture the behavior of polymeric particles in good solvent. In particular, all beads interact via a steric repulsion, modeled as a Weeks–Chandler–Anderson (WCA) potential

11 $$V_{WCA}\left(r\right)=\{\begin{array}{ll} 4\epsilon \left[{\left(\frac{\sigma }{r}\right)}^{12}-{\left(\frac{\sigma }{r}\right)}^{6}\right]+\epsilon , & \mathrm{if}\;r\leq 2^{1/6}\sigma \\ 0, & \mathrm{otherwise} \end{array}$$

with ϵ setting the energy scale and *r* the distance between two particles. Bonded beads also experience the finitely extensible nonlinear elastic (FENE) potential

12 $$V_{FENE}\left(r\right)=-\epsilon k_{F}{R_{0}}^{2}\ln\left[1-{\left(\frac{r}{R_{0}\sigma }\right)}^{2}\right]\;\mathrm{if}\;r<R_{0}\sigma$$

with *k*_F_ = 15 determining the stiffness of the bond and *R*_0_ = 1.5 the maximum bond distance. These equations are generalized by the standard mixed terms for unequal sizes when dealing with the core of the stars interacting with all other monomers.

In order to capture the affinity of the polymer to the solvent, we use the so-called solvophobic potential,^55^ commonly employed for PNIPAM–BIS microgels:^51^

13 $$V_{\alpha }\left(r\right)=\{\begin{array}{ll} -\epsilon \alpha , & \mathrm{if}r\leq 2^{1/6}\sigma \\ \frac{1}{2}\alpha \epsilon \left[\cos\left(\gamma {\left(r/\sigma \right)}^{2}+\beta \right)-1\right], & \mathrm{if}2^{1/6}\sigma <r<R_{0}\sigma \\ 0, & \mathrm{if}r>R_{0}\sigma \end{array}$$

with γ = π­(2.25 – 2^1/3^)^−1^, with β = 2π – 2.25γ. This effective attraction, implicitly modeling the solvent effect, is controlled by the solvophobic parameter α, which plays the role of an effective temperature. For α = 0, the microgel is maximally swollen and no attraction in present, while, upon increasing α, the microgel collapses. The VPT for this model occurs at α ∼ 0.65,^31,51^ and a linear mapping between α and the real temperature of the experiments has been established for PNIPAM–BIS microgels in refs 31 and 32 and also reported in the inset of Figure 6. We have further tested the variant in which EGDMA cross-linkers remain solvophilic at all temperatures, finding basically no difference in the results with respect to the case in which they are thermoresponsive. Therefore, for simplicity, we adopt the last modeling approach.

### Simulations and Calculated Quantities

Molecular dynamics simulations are performed in the NVT ensemble with a reduced constant temperature T* = *k*_B_ *T*/ϵ = 1, with *k*_B_ the Boltzmann constant and *T* the temperature, which is controlled by a Nosé-Hoover thermostat. The simulated macromolecule, either a star or a microgel, is placed in the center of the simulation box with side 400σ and periodic boundary conditions. Equilibration runs are carried out for at least 1 × 10^6^δ*t*, with δ*t* = 0.002τ and $\tau =\sqrt{m\sigma^{2}/\epsilon }$ the unit of time, followed by production runs for at least 10 × 10^6^δ*t*. All simulations are performed using the *LAMMPS* package.^56^

From the coordinates of all the beads, we then calculate the form factor *P*(**q**) as

$$P\left(q\right)=\left\langle \frac{1}{N}\overset{N}{\underset{i=1}{\sum }}\overset{N}{\underset{j=1}{\sum }}\exp\left[iq\cdot \left(r_{i}-r_{j}\right)\right]\right\rangle$$ 14

where *N* is the total number of monomers in the particle and the brackets denote an average over different configurations. Similarly, we also compute the total density profile ρ­(*r*) as a function of the distance *r* from the center of mass, as

15 $$\rho \left(r\right)=\left\langle \overset{N}{\underset{i=1}{\sum }}\delta \left(\left|\mathrm{r⃗}-{\mathrm{r⃗}}_{i}\right|\right)\right\rangle$$

The density profiles of the cross-linkers are calculated considering the sum in eq 15 only up to the number of cross-linkers *N*_c_. The density profiles closer to star conditions should be described by a *r* ^–4/3^ dependence at intermediate distances, according to the Daoud–Cotton model in the swollen regime.^5^ However, at large distances, there is always a more diffuse layer of polymers that is assumed to follow a Gaussian decay.^57^ Therefore, the density profiles are described according to the following functional form:^58^

16 $$\rho^{\mathrm{star}}\left(r\right)∼A_{1}r^{-4/3}f_{b}\left(r,r_{b}\right)+A_{2}\left[1-f_{b}\left(r,r_{b}\right)\right]\;\exp\left[-{\left(\frac{r-r_{1}}{r_{2}}\right)}^{2}\right]$$

with $f_{b}\left(r,r_{b}\right)=\exp\left[-{\left(\frac{r}{r_{b}}\right)}^{4}\right]$ a bridge function between the two regimes.

Finally, the radius of gyration *R*_g_, which gives a measure of the size of the particle, is calculated as

$$R_{g}=\left\langle {\left(\frac{1}{N}\overset{N}{\underset{i}{\sum }}{\left({\mathrm{r⃗}}_{i}-{\mathrm{r⃗}}_{cm}\right)}^{2}\right)}^{1/2}\right\rangle$$ 17

where $\overset{⃗}{r_{i}\;}$ refers to the position of the *i*th monomer, ${\mathrm{r⃗}}_{cm}$ to the center of mass of the star or of the microgel.

## Supplementary Material



## Notes

Additional data on the microgel characterization via DLS measurements, results for PNIPAM–EGDMA microgels with *C*_EGDMA_ = 1% synthesized with a different initiator, a comparison of the *C*_EGDMA_ = 10% microgel with the star polymer model, numerical density profiles for star polymers compared to star-like microgels, and additional results for the swelling behavior of *in silico* microgels (PDF)
