# Supplementary material for: Star-Like Thermoresponsive Microgels as an Emerging Class of Soft Nanocolloids
Source: ACS Nano. 2025 Sep 29;19(40):35447–58. doi: 10.1021/acsnano.5c08174 (PMC12530042; doi:10.1021/acsnano.5c08174)
Supplement: Supplementary file 1 [file nn5c08174_si_001.pdf]

Supporting Information Available

Star-like thermoresponsive microgels as an emerging class of soft nanocolloids

Elisa Ballin[1,2,†,*], Francesco Brasili[1,2,†], Tommaso Papetti[1,2], Jacopo Vialetto[3,4], Michael Sztucki[5], Simona Sennato[1,2], Marco Laurati[3,4], Emanuela Zaccarelli[1,2,*]

[1] Dipartimento di Fisica, Sapienza Università di Roma, Piazzale A. Moro 2, 00185 Roma, Italy

[2] CNR-ISC, Uos Sapienza, Piazzale A. Moro 2, 00185 Roma, Italy

[3] Dipartimento di Chimica "Ugo Schiff", Università di Firenze, Sesto Fiorentino (FI), 50019 Italy

[4] Consorzio per lo Sviluppo dei Sistemi a Grande Interfase (CSGI), via della Lastruccia 3, Sesto Fiorentino (FI), 50019, Italy

[5] European Synchrotron Radiation Facility – The European Synchrotron, 71 avenue des Martyrs F-38043 Grenoble, France

[†] These authors contributed equally

[*]Corresponding authors: elisa.ballin@uniroma1.it, marco.laurati@unifi.it, emanuela.zaccarelli@cnr.it

DLS characterisation

In Figure S1 we report the hydrodynamic radius as a function of temperature for microgels synthesized with different contents of EGDMA from 0.5% to 10%. We report also the curve for $C_{\mathrm{EGDMA}} = 1\%$ synthesized with APS as initiator. As discussed in Results section, microgels retain a high swelling capacity and a very sharp transition also at high crosslinker content.

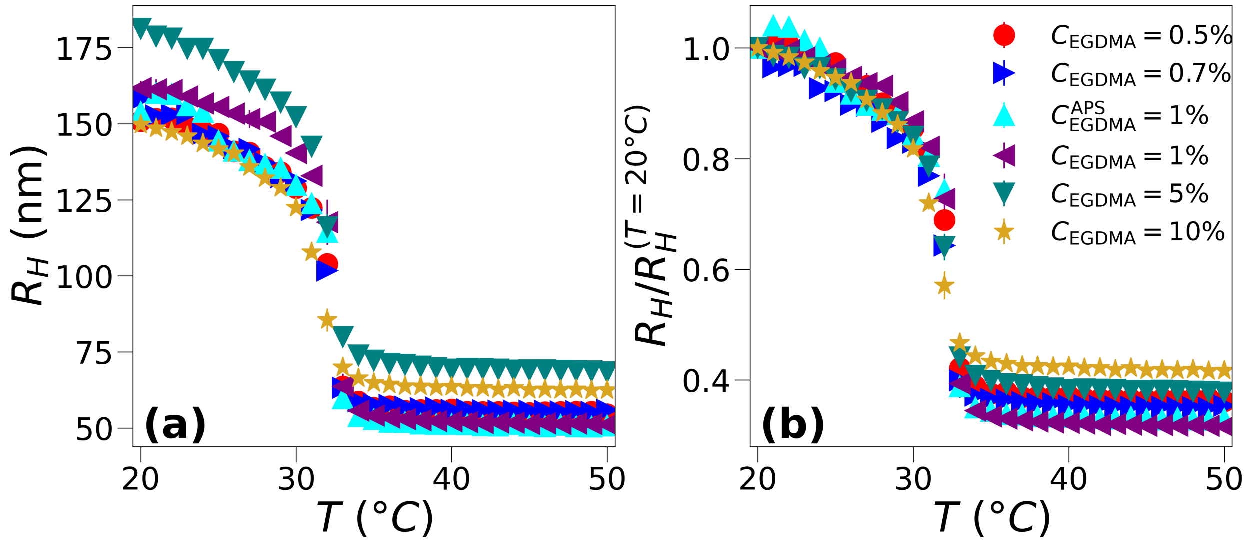

Figure S1: (a) Hydrodynamic radius as a function of temperature T of PNIPAM-EGDMA microgels synthesized with different content of EGDMA and initiators; (b) corresponding normalized curves.

In Table S1 we report the main characteristics of PNIPAM-EGDMA microgels as determined by DLS measurements.

Table S1: Values of the hydrodynamic radius at T = 25°C and T = 45°C, swelling ratio S_R, sharpness parameter s and VPT temperature T_c for microgels synthesized with C_{EGDMA} from 0.5%, to 10%.

C_{EGDMA} (%)	$R_H^{T=25°\mathrm{C}}$ (nm)	$R_H^{T=45°\mathrm{C}}$ (nm)	S_R	s (°C^{-1})	T_c (°C)
0.5	147 ± 2	55.0 ± 0.2	2.74 ± 0.04	1.3 ± 0.2	32.17 ± 0.07
0.7	146 ± 2	55.7 ± 0.2	2.84 ± 0.03	1.2 ± 0.2	32.09 ± 0.07
1	156 ± 2	51.3 ± 0.1	3.15 ± 0.05	1.5 ± 0.2	32.28 ± 0.08
5	171 ± 1	68.9 ± 0.3	2.63 ± 0.02	0.93 ± 0.09	31.97 ± 0.06
10	141.7 ± 0.5	62.5 ± 0.1	2.40 ± 0.01	0.66 ± 0.05	31.41 ± 0.03

Additional Results for microgels with $\mathbf{C_{EGDMA}} = 1\%$

In Fig. S2(a) we show that the low-T data for C_{EGDMA} = 1% are also well-described by the simple star model of Dozier and coworkers,[30] amounting to the sum of Eqns. 6 and 7. We find $R_g \sim 91$nm, $\mu \approx 0.66$ and $\xi \sim 20$nm and note that the parameters of the fit are

largely identical to those reported in the main text (Table 1) when using the star-like fuzzy sphere model of Eq. 3. However, at high T, the star model is not sufficient to describe the experimental form factors, which is why we resort to the more general model in all cases.

In addition, we test the possible influence of a different initiator on the resulting synthesis. To this aim, we also synthesized PNIPAM-EGDMA microgels with $C_{EGDMA} = 1\%$ using APS as initiator, using the same amounts of reagents reported in Table 2 and injecting 1.2 ml of a water solution containing 9.8 mg of APS to start the reaction. In Figure S2 (b), we show the form factors of these microgels at $T = 25$°C and $T = 45$°C. It can be seen that by varying the initiator, a similar structure to the KPS-initiated ones is obtained. At low temperature the microgels synthesized with APS display a small peak with respect to $C_{\mathrm{EGDMA}} = 1\%$-KPS microgel. This small difference probably is due to a greater incorporation of EGDMA that may vary from one synthesis to another. At high temperature the form factors of the two microgels are perfectly superimposed. The data at both temperatures are again well described by the star-like fuzzy sphere model of Eq. 3, with parameters reported in Table S2, very similar to the ones corresponding to the synthesis in the presence of KPS reported in Table 1 of the main text.

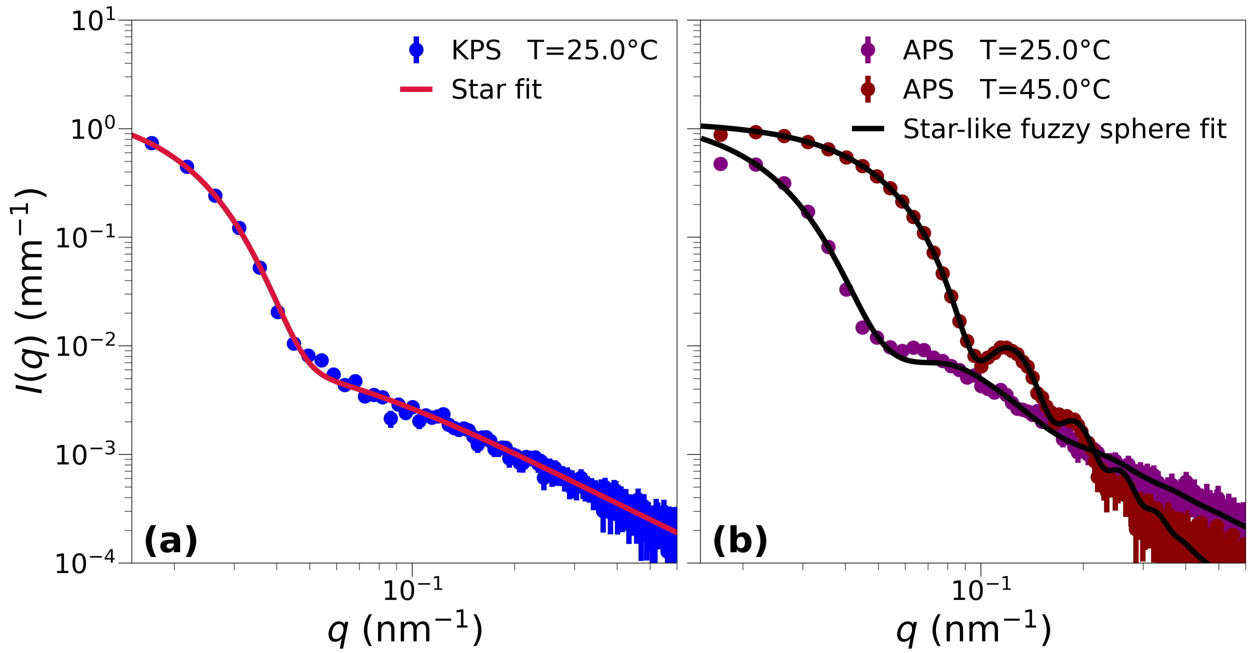

Figure S2: (a) SAXS scattering intensities I(Q) at $T = 25$°C for PNIPAM-EGDMA microgels with $C_{EGDMA} = 1\%$ as in Fig. 2(b) of the main text, but now fitted with the simple star model proposed by Dozier *et al.*;[30] (b) I(Q) for $C_{EGDMA} = 1\%$ microgels synthesized with APS as initiator at $T = 25$°C and $T = 45$°C. The lines are fits via the star-like fuzzy sphere model in Eq. 3, whose fit parameters are reported in Table S2.

Table S2: Best-fit parameters for $C_{\text{EGDMA}} = 1\%$ synthesized with APS as initiator derived from the star-like fuzzy sphere model (Eq. 3). $\Delta\rho^{cs}$ and $\Delta\rho^{s0}$ represent the difference between the scattering length densities between the core and the shell $(\rho_c - \rho_s)$ and between the shell and the solvent $(\rho_s - \rho_0\ )$, respectively.

T(°C)	R_c(nm)	t(nm)	σ_s(nm)	μ	ξ(nm)	$\Delta\rho^{cs}$(nm^{-2})	$\Delta\rho^{s0}$(nm^{-2})
25	24	64	28	0.66	20	0.1	0.009
45	25	21	4	1.77	14	0.009	0.04

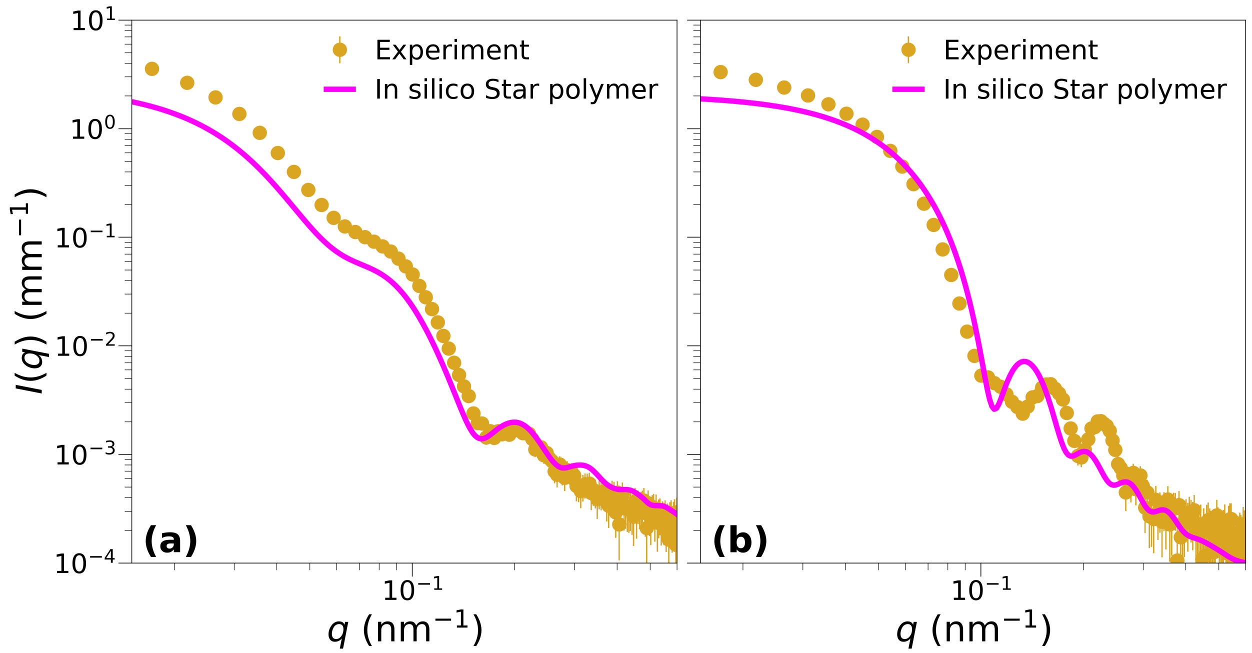

Figure S3: SAXS scattering intensities I(Q) at $T = 25$°C (a) and $T = 45$°C (b) for samples with $C_{\text{EGDMA}} = 10\%$. The solid lines represents the form factors of a simulated star-polymer at $\alpha = 0.0$ (a) and $\alpha = 0.8$ (b).

Density profiles for *in silico* star microgel and star polymer models

In Figure S4 we report the comparison between the density profiles of simulated $C_{\text{EGDMA}} = 1\%$ star-microgel and the corresponding star polymer with $f = 80$, $N_f = 200$ best reproducing the experimental data. A clear difference is present in the region of the core, since in the case of the star polymer, there is a single rigid core onto which polymer chains are attached. In the case of the star microgel the core is full of crosslinkers. However, the tail region is similar in both models, following Eq. 16 as shown in the main manuscript, corroborating that the proposed method for modelling these microgels shows the features of a star polymer.

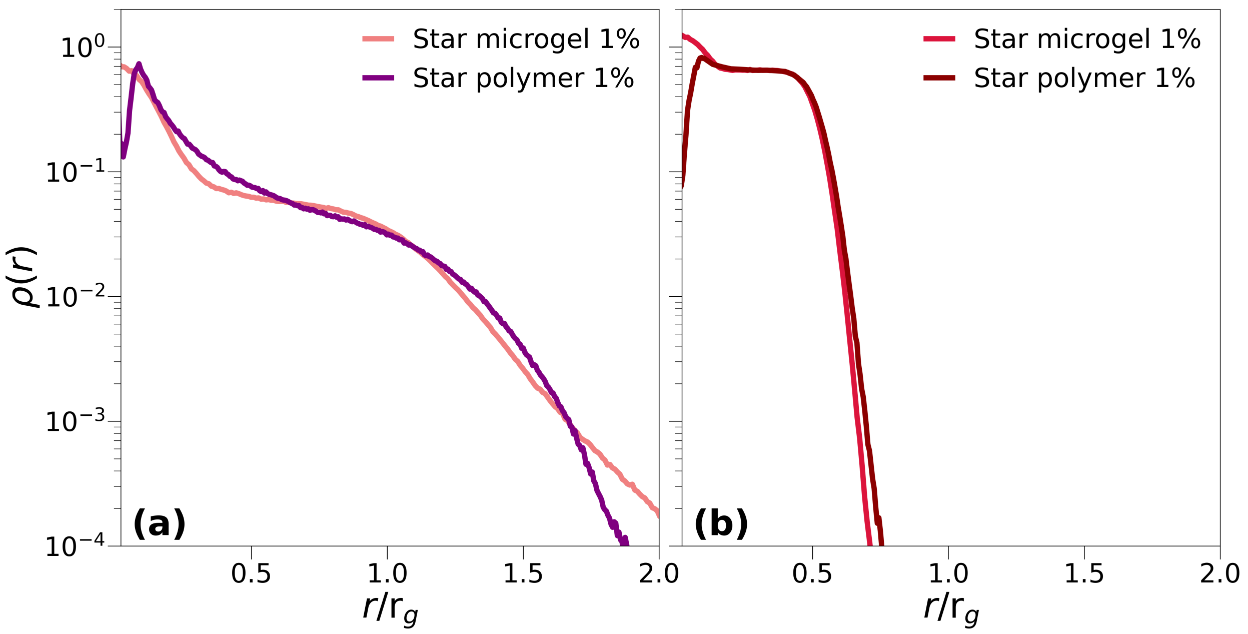

Figure S4: Comparison between the density profiles of simulated $C_{\mathrm{EGDMA}} = 1\%$ star-microgel and $f = 80$, $N_f = 200$ star-polymer at $\alpha = 0.0$ (a) and $\alpha = 0.8$ (b). The x-axis of each curve is rescaled by the respective radius of gyration calculated at $\alpha = 0.0$.

***In silico* swelling behaviour**

In Figure S5 we report the comparison between the swelling curves of simulated $C_{\mathrm{EGDMA}} = 1\%$ and 10% star-microgels and $C_{\mathrm{BIS}} = 1\%$ and 10% standard core-shell microgels. Unlike standard microgels, star-like ones retain a high swelling capacity even at high crosslinker content. Moreover the VPT remains very sharp if compared to the swelling curve of $C_{\mathrm{BIS}} = 10\%$, even by not taking into account the different α-temperature mapping that we established in the inset of Fig. 6(b).

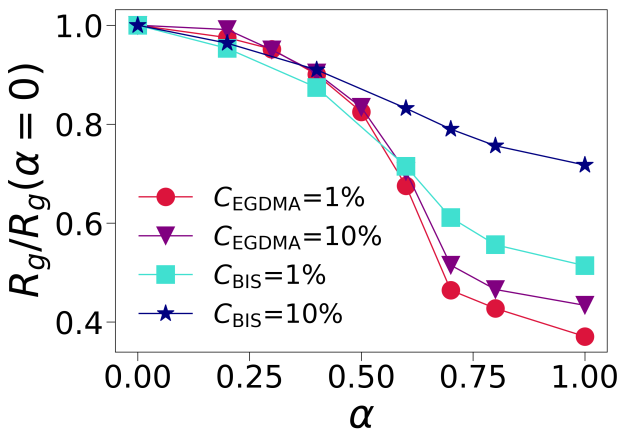

Figure S5: Comparison between swelling curves of simulated $C_{\mathrm{EGDMA}} = 1\%$ and 10% star-microgel and $C_{\mathrm{BIS}} = 1\%$ and 10% standard core-shell microgels.
